# Effectiveness of convalescent plasma therapy in severe COVID-19 patients

**DOI:** 10.1073/pnas.2004168117

**Published:** 2020-04-06

**Authors:** Kai Duan, Bende Liu, Cesheng Li, Huajun Zhang, Ting Yu, Jieming Qu, Min Zhou, Li Chen, Shengli Meng, Yong Hu, Cheng Peng, Mingchao Yuan, Jinyan Huang, Zejun Wang, Jianhong Yu, Xiaoxiao Gao, Dan Wang, Xiaoqi Yu, Li Li, Jiayou Zhang, Xiao Wu, Bei Li, Yanping Xu, Wei Chen, Yan Peng, Yeqin Hu, Lianzhen Lin, Xuefei Liu, Shihe Huang, Zhijun Zhou, Lianghao Zhang, Yue Wang, Zhi Zhang, Kun Deng, Zhiwu Xia, Qin Gong, Wei Zhang, Xiaobei Zheng, Ying Liu, Huichuan Yang, Dongbo Zhou, Ding Yu, Jifeng Hou, Zhengli Shi, Saijuan Chen, Zhu Chen, Xinxin Zhang, Xiaoming Yang

**Affiliations:** ^a^China National Biotec Group Company Limited, 100029 Beijing, China;; ^b^National Engineering Technology Research Center for Combined Vaccines, Wuhan Institute of Biological Products Co. Ltd., 430207 Wuhan, China;; ^c^First People’s Hospital of Jiangxia District, 430200 Wuhan, China;; ^d^Sinopharm Wuhan Plasma-derived Biotherapies Co., Ltd, 430207 Wuhan, China;; ^e^Key Laboratory of Special Pathogens, Wuhan Institute of Virology, Center for Biosafety Mega-Science, Chinese Academy of Sciences, 430071 Wuhan, China;; ^f^WuHan Jinyintan Hospital, 430023 Wuhan, China;; ^g^Department of Respiratory and Critical Care Medicine, Ruijin Hospital, Shanghai Jiao Tong University School of Medicine, 200025 Shanghai, China;; ^h^National Research Center for Translational Medicine, Ruijin Hospital, Shanghai Jiao Tong University School of Medicine, 200025 Shanghai, China;; ^i^Institute of Respiratory Diseases, Ruijin Hospital, Shanghai Jiao Tong University School of Medicine, 200025 Shanghai, China;; ^j^Clinical Research Center, Department of Gastroenterology, Ruijin Hospital North, Shanghai Jiao Tong University School of Medicine, 200018 Shanghai, China;; ^k^Wuhan Blood Center, 430030 Wuhan, China;; ^l^State Key Laboratory of Medical Genomics, Shanghai Institute of Hematology, National Research Center for Translational Medicine, Ruijin Hospital, Shanghai Jiao Tong University School of Medicine, 200025 Shanghai, China;; ^m^Research Laboratory of Clinical Virology, Ruijin Hospital and Ruijin Hospital North, National Research Center for Translational Medicine, Shanghai Jiao Tong University School of Medicine, 200025 Shanghai, China;; ^n^National Institute for Food and Drug Control of China, 102629 Beijing, China

**Keywords:** COVID-19, convalescent plasma, treatment outcome, pilot project

## Abstract

Currently, there are no approved specific antiviral agents for novel coronavirus disease 2019 (COVID-19). In this study, 10 severe patients confirmed by real-time viral RNA test were enrolled prospectively. One dose of 200 mL of convalescent plasma (CP) derived from recently recovered donors with the neutralizing antibody titers above 1:640 was transfused to the patients as an addition to maximal supportive care and antiviral agents. The primary endpoint was the safety of CP transfusion. The second endpoints were the improvement of clinical symptoms and laboratory parameters within 3 d after CP transfusion. The median time from onset of illness to CP transfusion was 16.5 d. After CP transfusion, the level of neutralizing antibody increased rapidly up to 1:640 in five cases, while that of the other four cases maintained at a high level (1:640). The clinical symptoms were significantly improved along with increase of oxyhemoglobin saturation within 3 d. Several parameters tended to improve as compared to pretransfusion, including increased lymphocyte counts (0.65 × 10^9^/L vs. 0.76 × 10^9^/L) and decreased C-reactive protein (55.98 mg/L vs. 18.13 mg/L). Radiological examinations showed varying degrees of absorption of lung lesions within 7 d. The viral load was undetectable after transfusion in seven patients who had previous viremia. No severe adverse effects were observed. This study showed CP therapy was well tolerated and could potentially improve the clinical outcomes through neutralizing viremia in severe COVID-19 cases. The optimal dose and time point, as well as the clinical benefit of CP therapy, needs further investigation in larger well-controlled trials.

## Significance

COVID-19 is currently a big threat to global health. However, no specific antiviral agents are available for its treatment. In this work, we explore the feasibility of convalescent plasma (CP) transfusion to rescue severe patients. The results from 10 severe adult cases showed that one dose (200 mL) of CP was well tolerated and could significantly increase or maintain the neutralizing antibodies at a high level, leading to disappearance of viremia in 7 d. Meanwhile, clinical symptoms and paraclinical criteria rapidly improved within 3 d. Radiological examination showed varying degrees of absorption of lung lesions within 7 d. These results indicate that CP can serve as a promising rescue option for severe COVID-19, while the randomized trial is warranted.

Since December 2019, a pneumonia associated with severe acute respiratory syndrome coronavirus 2 (SARS-CoV-2), named as coronavirus disease 2019 (COVID-19) by World Health Organization (WHO), emerged in Wuhan, China (1–3). The epidemic spread rapidly worldwide within 3 mo and was characterized as a pandemic by WHO on March 11, 2020. As of March 12, 2020, a total of 80,980 confirmed cases and 3,173 deaths had been reported in China. Meanwhile, a total of 44,377 confirmed cases and 1,446 deaths was reported in another 108 countries or regions. Currently, there are no approved specific antiviral agents targeting the novel virus, while some drugs are still under investigation, including remdesivir and lopinavir/ritonavir (4, 5). Although remdesivir was reported to possess potential antiviral effect in one COVID-19 patient from the United States, randomized controlled trials of this drug are ongoing to determine its safety and efficacy (6). Moreover, the corticosteroid treatment for COVID-19 lung injury remains controversial, due to delayed clearance of viral infection and complications (7, 8). Since the effective vaccine and specific antiviral medicines are unavailable, it is an urgent need to look for an alternative strategy for COVID-19 treatment, especially among severe patients.

Convalescent plasma (CP) therapy, a classic adaptive immunotherapy, has been applied to the prevention and treatment of many infectious diseases for more than one century. Over the past two decades, CP therapy was successfully used in the treatment of SARS, MERS, and 2009 H1N1 pandemic with satisfactory efficacy and safety (9–12). A meta-analysis from 32 studies of SARS coronavirus infection and severe influenza showed a statistically significant reduction in the pooled odds of mortality following CP therapy, compared with placebo or no therapy (odds ratio, 0.25; 95% confidence interval, 0.14–0.45) (13). However, the CP therapy was unable to significantly improve the survival in the Ebola virus disease, probably due to the absence of data of neutralizing antibody titration for stratified analysis (14). Since the virological and clinical characteristics share similarity among SARS, Middle East Respiratory Syndrome (MERS), and COVID-19 (15), CP therapy might be a promising treatment option for COVID-19 rescue (16). Patients who have recovered from COVID-19 with a high neutralizing antibody titer may be a valuable donor source of CP. Nevertheless, the potential clinical benefit and risk of convalescent blood products in COVID-19 remains uncertain. Hence, we performed this pilot study in three participating hospitals to explore the feasibility of CP treatment in 10 severe COVID-19 patients.

## Results

### Neutralizing Activity of CP against SARS-CoV-2.

The neutralizing activity against SARS-CoV-2 was evaluated by classical plaque reduction test using a recently isolated viral strain (1). Among the first batch of CP samples from 40 recovered COVID-19 patients, 39 showed high antibody titers of at least 1:160, whereas only one had an antibody titer of 1:32. This result laid the basis for our pilot clinical trial using CP in severe patients.

### General Characteristics of Patients in the Trial.

From January 23, 2020, to February 19, 2020, 10 severe COVID-19 patients (six males and four females) were enrolled and received CP transfusion. The median age was 52.5 y (interquartile range [IQR], 45.0 y to 59.5 y) (Table 1). None of the patients had direct exposure to Huanan Seafood Wholesale Market. The median time from onset of symptoms to hospital admission and CP transfusion was 6 d (IQR, 2.5 d to 8.5 d) and 16.5 d (IQR, 11.0 d to 19.3 d), respectively. Three patients were affected by clustering infection. The most common symptoms at disease onset were fever (7 of 10 patients), cough (eight cases), and shortness of breath (eight cases), while less common symptoms included sputum production (five cases), chest pain (two cases), diarrhea (two cases), nausea and vomiting (two cases), headache (one case), and sore throat (one case). Four patients had underlying chronic diseases, including cardiovascular and/or cerebrovascular diseases and essential hypertension. Nine patients received arbidol monotherapy or combination therapy with remdesivir (in one case not included in the current clinical trial), or ribavirin, or peramivir, while one patient received ribavirin monotherapy (Table 2). Antibacterial or antifungal treatment was used when patients had coinfection. Six patients received intravenous (i.v.) methylprednisolone (20 mg every 24 h).

**Table 1.** Clinical characteristics of patients receiving CP transfusion

Patient no.	Sex	Age, y	Clinical classification	Days of admission from symptom onset	Days of CP therapy from symptom onset	Clustering infection	Principal symptoms	Comorbidity
1	M	46	Severe	8	11	No	Fever, cough, sputum production, shortness of breath, chest pain	Hypertension
2	F	34	Severe	0	11	Yes	Cough, shortness of breath, chest pain, nausea and vomiting	None
3	M	42	Severe	8	19	Yes	Fever, cough, sputum production, shortness of breath, sore throat, diarrhea	Hypertension
4	F	55	Severe	10	19	No	Fever, cough, sputum production, shortness of breath	None
5	M	57	Severe	4	14	No	Fever, shortness of breath	None
6	F	78	Severe	8	17	Yes	Fever, cough, sputum production, shortness of breath, muscle ache	None
7	M	56	Severe	4	16	No	Fever, cough, sputum production, arthralgia	None
8	M	67	Severe	10	20	No	Fever, cough, headache, diarrhea, vomiting	Cardiovascular and cerebrovascular diseases
9	F	49	Severe	1	10	No	Cough, shortness of breath	None
10	M	50	Severe	3	20	No	Shortness of breath	Hypertension

M, male; F, female.

**Table 2.** Other treatments of ten patients receiving CP transfusion

	Drugs administered			Oxygen support	
Patient no.	Antiviral treatment	Antibiotic or antifungal treatment	Corticosteroids treatment	Before CP therapy	After CP therapy
1	Arbidol 0.2 g q8h po.Ribavirin 0.5 g qd i.v.	Cefoperazone Sodium i.v.	None	High-flow nasal cannula, mechanical ventilation	Mechanical ventilation
2	Arbidol 0.2 g q8h po.	Cefoperazone Sodium i.v.	None	None	None
3	Arbidol 0.2 g q8h po.	Moxifloxacin i.v.	Methylprednisolone i.v.	High-flow nasal cannula, mechanical ventilation	High-flow nasal cannula
4	Ribavirin 0.5 g qd i.v.	Linezolid i.v.Imipenem-Sitastatin Sodium i.v.	Methylprednisolone i.v.	Mechanical ventilation	High-flow nasal cannula
5	Arbidol 0.2 g q8h po.	Moxifloxacin i.v.	Methylprednisolone i.v.	Low-flow nasal cannula	Low-flow nasal cannula
	Remdesivir 0.2 g qd i.v.	Cefoperagone Sodium and Tazobactam Sodium i.v.			
	IFN-ɑ 500MIU qd inh.				
6	Arbidol 0.2 g q8h po.	Cefoperazone Sodium i.v.Levofloxacin i.v.	Methylprednisolone i.v.	High-flow nasal cannula	High-flow nasal cannula
7	Arbidol 0.2 g q8h po.	Cefoperagone Sodium and Tazobactam Sodium i.v.	Methylprednisolone i.v.	High-flow nasal cannula	None
		Fluconazole i.v.			
8	Arbidol 0.2 g q8h po.	None	None	None	None
	Ribavirin 0.5 g qd i.v.				
9	Arbidol 0.2 g q8h po.Oseltamivir 75 mg q12h po.	None	None	Low-flow nasal cannula	Low-flow nasal cannula (Intermittent)
	Peramivir 0.3 g qd i.v.				
10	Arbidol 0.2 g q8h po.IFN-ɑ 500 MIU qd inh.	Cefoperazone Sodium i.v.Caspofungin i.v.	Methylprednisolone i.v.	High-flow nasal cannula	High-flow nasal cannula

po., per os; i.v., i.v. injection; inh., inhalation; q8h, every 8 h; qd, per day; q12h, every 12 h; MIU, million IU.

On computer-assisted tomography (CT), all patients presented bilateral ground-glass opacity and/or pulmonary parenchymal consolidation with predominantly subpleural and bronchovascular bundles distribution in the lungs. Seven patients had multiple lobe involvement, and four patients had interlobular septal thickening.

### Effects of CP Transfusion.

#### Improvement of clinical symptoms.

All symptoms in the 10 patients, especially fever, cough, shortness of breath, and chest pain, disappeared or largely improved within 1 d to 3 d upon CP transfusion. Prior to CP treatment, three patients received mechanical ventilation, three received high-flow nasal cannula oxygenation, and two received conventional low-flow nasal cannula oxygenation. After treatment with CP, two patients were weaned from mechanical ventilation to high-flow nasal cannula, and one patient discontinued high-flow nasal cannula. Besides, in one patient treated with conventional nasal cannula oxygenation, continuous oxygenation was shifted to intermittent oxygenation (Table 2).

#### Reduction of pulmonary lesions on chest CT examinations.

**Fig. 1.**
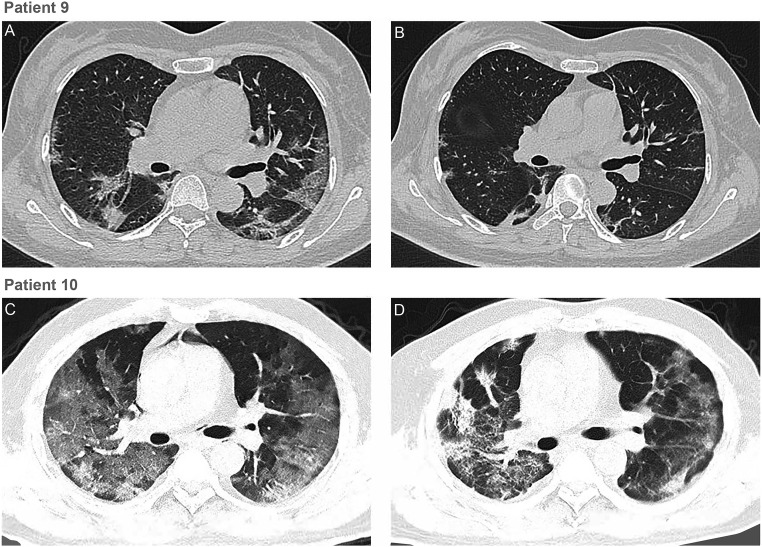
Chest CTs of two patients. (*A*) Chest CT of patient 9 obtained on February 9 (7 dpoi) before CP transfusion (10 dpoi) showed ground-glass opacity with uneven density involving the multilobal segments of both lungs. The heart shadow outline was not clear. The lesion was close to the pleura. (*B*) CT Image of patient 9 taken on February 15 (13 dpoi) showed the absorption of bilateral ground-glass opacity after CP transfusion. (*C*) Chest CT of patient 10 was obtained on February 8 (19 dpoi) before CP transfusion (20 dpoi). The brightness of both lungs was diffusely decreased, and multiple shadows of high density in both lungs were observed. (*D*) Chest CT of patient 10 on February 18 (29 dpoi) showed those lesions improved after CP transfusion.

According to chest CTs, all patients showed different degrees of absorption of pulmonary lesions after CP transfusion. Representative chest CT images of patient 9 and patient 10 are shown on Fig. 1. Patient 9, a 49-y-old female admitted 1 day postonset of illness (dpoi), showed the most obvious pulmonary image improvement. At 10 dpoi, one dose of 200-mL transfusion of CP was given. The SARS-CoV-2 RNA converted to negative at 12 dpoi. Compared with the result at 7 dpoi, massive infiltration and ground-glass attenuation disappeared on CT image performed at 13 dpoi, accompanied by a much better pulmonary function. Patient 10, a 50-y-old male, was admitted 3 dpoi and was given a 200-mL transfusion of CP at 20 dpoi. His chest CT presented massive infiltration and widespread ground-glass attenuation on admission and started to show a gradual absorption of lung lesions 5 d after CP transfusion. The SARS-CoV-2 RNA became negative at 25 dpoi.

#### Amelioration of routine laboratory criteria and pulmonary function.

**Fig. 2.**
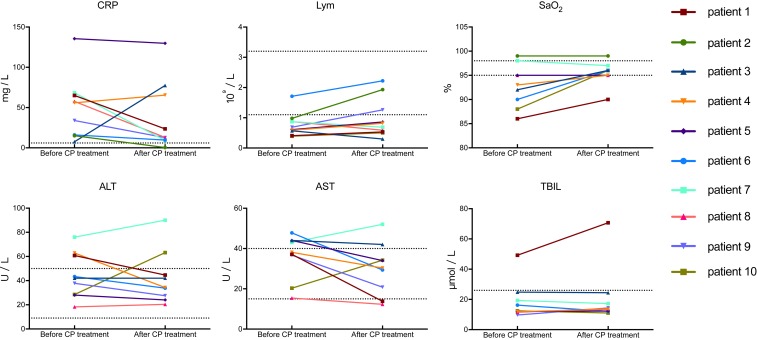
Dynamic changes of laboratory parameters in all patients. The dotted horizontal line represents the reference value range. SaO_2_, oxyhemoglobin saturation; TBIL, total bilirubin; ALT, alanine aminotransferase; AST, aspartate aminotransferase; Lym, lymphocyte.

Lymphocytopenia, an important index for prognosis in COVID-19 (2), tended to be improved after CP transfusion (median: 0.65 × 10^9^ per L vs. 0.76 × 10^9^ per L), 7 out of 10 patients showing an increase of lymphocyte counts (Fig. 2). Concerning other laboratory tests, we observed a tendency of decrement of parameters indicative of inflammation and/or liver dysfunction as compared to the status before CP therapy. These included C-reactive protein (CRP) (median: 55.98 mg/L vs. 18.13 mg/L), alanine aminotransferase (median: 42.00 U/L vs. 34.30 U/L), and aspartate aminotransferase (median: 38.10 U/L vs. 30.30 U/L) (Table 3). The total bilirubin (median: 12.40 μmol/L vs. 13.98 μmol/L) remained unchanged, except for an obvious increment in patient 1 (Fig. 2). An increase of SaO_2_ (median: 93.00% vs. 96.00%), a measurement constantly performed in most patients in our trial, was found, which could indicate recovering lung function. This temporal relationship was notable despite the provision of maximal supportive care and antiviral agents.

**Table 3.** Comparison of laboratory parameters before and after CP transfusion

Clinical factors	Before CP transfusion	After CP transfusion
CRP (mg/L, normal range 0 to 6)	55.98 (15.57 to 66.67)	18.13 (10.92 to 71.44)
Lymphocyte (10^9^ per L, normal range 1.1 to 3.2)	0.65 (0.53 to 0.90)	0.76 (0.52 to 1.43)
Alanine aminotransferase (U/L, normal range 9 to 50)	42.00 (28.25 to 61.85)	34.30 (25.75 to 53.90)
Aspartate aminotransferase (U/L, normal range 15 to 40)	38.10 (28.50 to 44.00)	30.30 (17.30 to 38.10)
Total bilirubin (μmol/L, normal range 0 to 26)	12.40 (11.71 to 22.05)	13.98 (12.20 to 20.80)
SaO_2_ (%, normal range ≥ 95)	93.00 (89.00 to 96.50)	96.00 (95.00 to 96.50)

SaO_2_, oxyhemoglobin saturation.

**Fig. 3.**
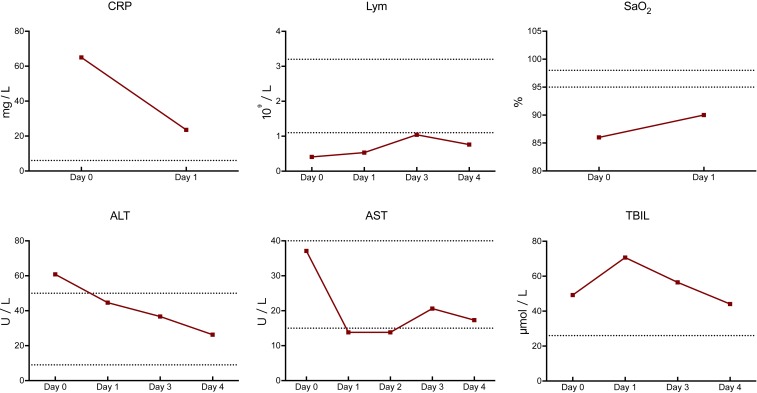
Change of laboratory parameters in patient 1. The *x* axis represents the day post-CP transfusion. The dotted horizontal line represents the reference value range.

Remarkably, patient 1, a 46-y-old male admitted 8 dpoi, had a very quick recovery, with much improved result of laboratory tests. He received antiviral drugs (arbidol and ribavirin) treatment and high-flow nasal cannula on admission. Mechanical ventilation was given at 10 dpoi for critical care support. CP transfusion was performed at 11 dpoi. At 12 dpoi, the SARS-CoV-2 test turned to negative, with a sharp decrease of CRP from 65.04 mg/L to 23.57 mg/L and increment of SaO_2_ from 86% to 90% (Fig. 3). The mechanical ventilation was successfully weaned off 2 d after CP transfusion. At 15 dpoi, a steady elevation of lymphocyte count and a drop of aminopherase level were observed, indicating improvement of immunological and hepatic function.

#### Increase of neutralizing antibody titers and disappearance of SARS-CoV-2 RNA.

We determined neutralizing antibody titers before and after CP transfusion in all patients except one (patient 2) (Table 4). The neutralizing antibody titers of five patients increased and four patients remained at the same level after CP transfusion. SARS-CoV-2 RNA, assayed by RT-PCR, was positive in seven patients and negative in three cases before CP transfusion. Of note is that SARS-CoV-2 RNA was decreased to an undetectable level in three patients on day 2, three patients on day 3, and one patient on day 6 after CP therapy. These results are in support of a neutralizing effect of CP on serum SARS-CoV-2.

**Table 4.** Comparison of serum neutralizing antibody titers and SARS-CoV-2 RNA load before and after CP therapy

Patient no.	CP transfusion date	Before CP transfusion			After CP transfusion		
		Date	Serum neutralizing antibody titers	Serum SARS-CoV-2 RNA load (Ct value)	Date	Serum neutralizing antibody titers	Serum SARS-CoV-2 RNA load (Ct value)
1	February 9	February 8	1:160	37.25	February 10	1:640	Negative
2	February 9	February 8	Unavailable	35.08	February 11	Unavailable	Negative
3	February 13	February 12	1:320	38.07	February 14	1:640	Negative
4	February 13	February 12	1:160	37.68	February 14	1:640	Negative
5	February 12	February 11	1:640	Negative	February 14	1:640	Negative
6	February 12	February 11	1:640	Negative	February 14	1:640	Negative
7	February 12	February 11	1:320	34.64	February 14	1:640	Negative
8	February 12	February 11	1:640	35.45	February 14	1:640	Negative
9	February 12	February 11	1:160	Negative	February 14	1:640	Negative
10	February 9	February 8	1:640	38.19	February 14	1:640	Negative

#### Outcome of patients treated with CP as compared to a recent historic control group.

A historic control group was formed by random selection of 10 patients from the cohort treated in the same hospitals and matched by age, gender, and severity of the diseases to the 10 cases in our trial. Baseline characteristics of patients between CP treatment group and control group showed no significant differences, while clinical outcomes of these two groups were different: three cases discharged while seven cases in much improved status and ready for discharge in CP group, as compared to three deaths, six cases in stabilized status, and one case in improvement in the control group (*P* < 0.001; *SI Appendix*, Table S1).

### Adverse Effects of CP Transfusions.

Patient 2 showed an evanescent facial red spot. No serious adverse reactions or safety events were recorded after CP transfusion.

## Discussion

Our study explores the feasibility of CP therapy in COVID-19. All enrolled severe COVID-19 patients achieved primary and secondary outcomes. One dose of 200-mL CP transfusion was well tolerated, while the clinical symptoms significantly improved with the increase of oxyhemoglobin saturation within 3 d, accompanied by rapid neutralization of viremia.

Severe pneumonia caused by human coronavirus was characterized by rapid viral replication, massive inflammatory cell infiltration, and elevated proinflammatory cytokines or even cytokine storm in alveoli of lungs, resulting in acute pulmonary injury and acute respiratory distress syndrome (ARDS) (17). Recent studies on COVID-19 demonstrated that the lymphocyte counts in the peripheral blood were remarkably decreased and the levels of cytokines in the plasma from patients requiring intensive care unit (ICU) support, including IL-6, IL-10, TNF-ɑ, and granulocyte-macrophage colony-stimulating factor, were significantly higher than in those who did not require ICU conditions (2, 18). CP, obtained from recovered COVID-19 patients who had established humoral immunity against the virus, contains a large quantity of neutralizing antibodies capable of neutralizing SARS-CoV-2 and eradicating the pathogen from blood circulation and pulmonary tissues (19). In the present study, all investigated patients achieved serum SARS-CoV-2 RNA negativity after CP transfusion, accompanied by an increase of oxygen saturation and lymphocyte counts, and the improvement of liver function and CRP. The results suggest that the inflammation and overreaction of the immune system were alleviated by antibodies contained in CP. The case fatality rates (CFRs) in the present study were 0% (0/10), which was comparable to the CFRs in SARS, which varied from 0% (0/10) to 12.5% (10/80) in four noncomparative studies using CP treatment (9, 20–22). Based on our preliminary results, CP therapy can be an easily accessible, promising, and safe rescue option for severe COVID-19 patients. It is, nevertheless, worth mentioning that the absorption of pulmonary lesions often lagged behind the improvement of clinical symptoms, as shown in patients 9 and 10 in this trial.

The first key factor associated with CP therapy is the neutralizing antibody titer. A small sample study in MERS-CoV infection showed that the neutralizing antibody titer should exceed 1:80 to achieve effective CP therapy (12). To find eligible donors who have high levels of neutralizing antibody is a prerequisite. Cao et al. (23) showed that the level of specific neutralizing antibody to SARS-CoV decreased gradually 4 mo after the disease process, reaching undetectable levels in 25.6% (IgG) and 16.1% (neutralizing antibodies) of patients at 36 mo after disease status. A study from the MERS-CoV−infected patients and the exposed healthcare workers showed that the prevalence of MERS-CoV IgG seroreactivity was very low (2.7%), and the antibodies titer decreased rapidly within 3 mo (24). These studies suggested that the neutralizing antibodies represented short-lasting humoral immune response, and plasma from recently recovered patients should be more effective. In the present study, recently recovered COVID-19 patients, who were infected by SARS-CoV-2 with neutralizing antibody titer above 1:640 and recruited from local hospitals, should be considered as suitable donors. The median age of donors was lower than that of recipients (42.0 y vs. 52.5 y). Among the nine cases investigated, the neutralizing antibody titers of five patients increased to 1:640 within 2 d, while four patients kept the same level. The antibody titers in CP in COVID-19 seem thus higher than those used in the treatment of MERS patient (1:80) (12).

The second key factor associated with efficacy is the treatment time point. A better treatment outcome was observed among SARS patients who were given CP before 14 dpoi (58.3% vs. 15.6%; *P* < 0.01), highlighting the importance of timely rescue therapy (9). The mean time from onset of illness to CP transfusion was 16.5 d. Consistent with previous research, all three patients receiving plasma transfusion given before 14 dpoi (patients 1, 2, and 9) in our study showed a rapid increase of lymphocyte counts and a decrease of CRP, with remarkable absorption of lung lesions in CT. Notably, patients who received CP transfusion after 14 dpoi showed much less significant improvement, such as patient 10. However, the dynamics of the viremia of SARS-CoV-2 was unclear, so the optimal transfusion time point needs to be determined in the future.

In the present study, no severe adverse effects were observed. One of the risks of plasma transfusion is the transmission of the potential pathogen. Methylene blue photochemistry was applied in this study to inactivate the potential residual virus and to maintain the activity of neutralizing antibodies as much as possible, a method known to be much better than ultraviolet (UV) C light (25). No specific virus was detected before transfusion. Transfusion-related acute lung injury was reported in an Ebola virus disease woman who received CP therapy (26). Although uncommon in the general population receiving plasma transfusion, this specific adverse reaction is worth noting, especially among critically ill patients experiencing significant pulmonary injury (27). Another rare risk worth mentioning during CP therapy is antibody-dependent infection enhancement, occurring at subneutralizing concentrations, which could suppress innate antiviral systems and thus could allow logarithmic intracellular growth of the virus (28). The special infection enhancement also could be found in SARS-CoV infection in vitro (29). No such pulmonary injury and infection enhancement were observed in our patients, probably owing to high levels of neutralizing antibodies, timely transfusion, and appropriate plasma volume.

There were some limitations to the present study. First, except for CP transfusion, the patients received other standard care. All patients received antiviral treatment despite the uncertainty of the efficacy of drugs used. As a result, the possibility that these antiviral agents could contribute to the recovery of patients, or synergize with the therapeutic effect of CP, could not be ruled out. Furthermore, some patients received glucocorticoid therapy, which might interfere with immune response and delay virus clearance. Second, the median time from onset of symptoms to CP transfusion was 16.5 d (IQR, 11.0 d to 19.3 d). Although the kinetics of viremia during natural history remains unclear, the relationship between SARS-CoV-2 RNA reduction and CP therapy, as well as the optimal concentration of neutralizing antibodies and treatment schedule, should be further clarified. Third, the dynamic changes of cytokines during treatment were not investigated. Nevertheless, the preliminary results of this trial seem promising, justifying a randomized controlled clinical trial in a larger patient cohort.

In conclusion, this pilot study on CP therapy shows a potential therapeutic effect and low risk in the treatment of severe COVID-19 patients. One dose of CP with a high concentration of neutralizing antibodies can rapidly reduce the viral load and tends to improve clinical outcomes. The optimal dose and treatment time point, as well as the definite clinical benefits of CP therapy, need to be further investigated in randomized clinical studies.

## Materials and Methods

### Patients.

From January 23, 2020, to February 19, 2020, 10 patients in three participating hospitals (Wuhan Jinyintan Hospital; the Jiangxia District Hospital of Integrative Traditional Chinese and Western Medicine, Wuhan; and the First People’s Hospital of Jiangxia District, Wuhan) were recruited in this pilot study. All patients were diagnosed as having severe COVID-19 according to the WHO Interim Guidance (30) and the Guideline of Diagnosis and Treatment of COVID-19 of National Health Commission of China (version 5.0) (31), with confirmation by real-time RT-PCR assay. The enrollment criteria were one of the conditions 2 to 4 plus condition 1: 1) age ≥ 18 y; 2) respiratory distress, RR ≥30 beats/min; 3) oxygen saturation level less than 93% in resting state; and 4) partial pressure of oxygen (PaO_2_)/oxygen concentration (FiO_2_) ≤ 300 mmHg (1 mmHg = 0.133 kPa). The exclusion criteria were as follows: 1) previous allergic history to plasma or ingredients (sodium citrate); 2) cases with serious general conditions, such as severe organ dysfunction, who were not suitable for CP transfusion. Written informed consent according to the Declaration of Helsinki was obtained from each patient or legal relatives. This study was approved by the Ethics Committee of the China National Biotec Group Co., Ltd. (Approval number 2020-0001). The registration number of this trial is ChiCTR2000030046.

### Donors for Convalescent Plasma Transfusion.

Ten donor patients who recovered from COVID-19 were recruited from three participating hospitals. The recovery criteria were as follows: 1) normality of body temperature for more than 3 d, 2) resolution of respiratory tract symptoms, and 3) two consecutively negative results of sputum SARS-CoV-2 by RT-PCR assay (1-d sampling interval). The donor’s blood was collected after 3 wk postonset of illness and 4 d postdischarge. Written informed consent was obtained from each patient.

### Plasma Preparation Procedure and Quality Control.

Apheresis was performed using a Baxter CS 300 cell separator (Baxter). Convalescence plasma for treatment was collected from 40 donors. The median age was 42.0 y (IQR, 32.5 y to 49 y). A 200- to 400-mL ABO-compatible plasma sample was harvested from each donor depending on age and body weight, and each sample was divided and stored as 200-mL aliquots at 4 °C without any detergent or heat treatment. The CP was then treated with methylene blue and light treatment for 30 min in the medical plasma virus inactivation cabinet (Shandong Zhongbaokang Medical Appliance Co., Ltd).

### Serology Test and Real-Time RT-PCR Detection of SARS-CoV-2 and Other Pathogens.

The neutralizing activity of plasma was determined by plaque reduction neutralization test using SARS-CoV-2 virus in the high biosafe level (BSL-4) laboratory of Wuhan Institute of Virology, Chinese Academy of Sciences. Neutralization titer was defined as the highest serum dilution with 50% reduction in the number of plaques, as compared with the number of plaques in wells in the absence of novel coronavirus antibody as blank control. The neutralizing activity of the receptor-binding domain of antibody in the CP was detected by a sandwich enzyme-linked immunosorbent assay (ELISA). SARS-CoV-2 IgG antibody titer was tested by ELISA. SARS-CoV-2 RNA was detected by RT-PCR assay, and the result was presented as cycle threshold (Ct) value (Shanghai BioGerm Medical Biotechnology Co., Ltd). Methylene blue residue was detected by the verified UV method. The serology screening for hepatitis B and C virus, HIV, and syphilis spirochete was negative. The protocols for neutralization assay, serological test, and real-time RT-PCR detection of SARS-CoV RNA are presented in *SI Appendix*.

### Treatment.

All patients were admitted to the ICU and received antiviral therapy and other supportive care, while some patients received antibiotic treatment, antifungal treatment, glucocorticoid, and oxygen support at the appropriate situation. One dose of 200 mL of inactivated CP with neutralization activity of >1:640 was transfused into the patients within 4 h following the WHO blood transfusion protocol.

### Data Collection.

Clinical information of all enrolled patients was retrieved from the hospital electronic history system, including the baseline demographic data, days of illness duration, presenting symptoms, different kinds of examination, and methods of treatment. Bacterial coinfection was identified by a positive culture from respiratory, urinary, or blood culture within 48 h of hospital admission. Complications, including acute renal failure, acute coronary syndrome, myocarditis, ARDS, and nosocomial infection, were recorded. The applications of assisted mechanical ventilation, intranasal oxygen inhalation, and medication regimen were recorded. The SARS-CoV-2 RNA from the serum sample was monitored during treatment.

### Outcome Measures and Definitions.

The clinical symptoms were recorded by attending physicians daily. The blood test and biochemical tests were carried out every 1 d to 2 d. SARS-CoV-2 RNA was detected every 2 d to 3 d. CT scan was repeated every 3 d to 5 d. The primary endpoint was the safety of CP transfusion. The second endpoints were the improvement of clinical symptoms and laboratory and radiological parameters within 3 d after CP transfusion. Clinical symptoms improvement was defined as temperature normalization, relief of dyspnea, and oxygen saturation normalization, and radiological improvement was defined as different degrees of absorption of lung lesions.

### Statistical Analysis.

Continuous variables were presented as the median and IQR. Graphs were plotted using GraphPad Prism 7.0. Statistical software used included SPSS 24.0.

### Data Availability Statement.

All data relevant to this manuscript and available to the authors at the time of publication are included in the main text or *SI Appendix*. Further detailed data on patients that support the findings of this study have been deposited in the Open Science Framework (https://osf.io/gahz5).

## Supplementary Material

Supplementary File
