# Supplementary material for: Effectiveness of convalescent plasma therapy in severe COVID-19 patients
Source: Proc Natl Acad Sci U S A. 2020 Apr 6;117(17):9490–6. doi: 10.1073/pnas.2004168117 (PMC7196837; doi:10.1073/pnas.2004168117)
Supplement: Supplementary File [file pnas.2004168117.sapp.pdf]

Supplementary Materials

Neutralization assay

The virus neutralization test was carried out in a 12-well plate. The patient serum samples were heat-inactivated by incubation at 56 ℃ for 30 min before use. The serum samples (5 μL) were diluted to 1:10, 1:20, 1:40 or 1:80, and then an equal volume of virus stock(180 pfu/well) was added and incubated at 37℃ for 60 min in a 5% CO_2 incubator. After incubation, 500 μL mixtures were inoculated onto monolayer Vero E6 cells in a 48-well plate for 1 hour. Each serum was detected in triplicate. After removing the supernatant, the plate was washed twice with DMEM medium, then was added 1 mL of 0.9% methylcellulose-containing maintenance medium and incubated the cells in humidified incubator at 37 °C, 5% CO_2 for 3-5 days. Once plaques were developed in wells and observed by naked eye, and then cells were fixed with 8 % formaldehyde for 60 min at room temperature. Cells were stained with 6×methylene blue solution for more than 1 h. Neutralization titer was defined as the highest serum dilution with 50 % reduction in the number of plaques, as compared with the number of plaques in wells in the absence of novel coronavirus antibody as blank control.

www.pnas.org/cgi/doi/10.1073/pnas.2004168117

Serological test

Anti-COVID-19 IgG in human serum were determined by a sandwich ELISA according to the following protocol: (1) coat the wells with 100 μL of purified 2019-nCoV RBD protein with 100 ng/well in coating buffer at 4 °C overnight; (2) wash plates three times to remove unbound RBD protein , block each well with 200 μL of 0.05% skim milk and incubate plates at 37 °C for 90 min; (3) wash plates, add 100 μL of ten-fold serial diluted human serum into each well, and incubate at 37 °C for 60 min; (4) wash plates, add 100 μL of anti-Human IgG-HRP conjugated monoclonal antibody at a 1:5000 dilution in PBS to each well and incubate plates at 37 °C for 30 min; (5) wash plates five times, add 100 μL of TMB solution; (6) incubate plates at 37 °C for 15 min, stop reaction by the addition of 50 μL of 2 M H_2SO_4 ,and read OD value at 450 nm and 630nm. The OD cutoff was calculated by the equation, OD=0.093+Mean OD (blank control). Sample OD value was ≥cutoff OD value，indicating novel coronavirus neutralization IgG antibody positive.

Real-time RT–PCR detection of SARS-CoV-2 RNA

Serum samples were collected from patients for viral RNA extraction using QIAamp viral RNA mini kit (Qiagen, USA). The RNA template was used for real-time reverse transcription polymerase chain reaction (RT-PCR) assay of 2019-nCoVRNA. Two target genes, including open reading frame1ab (ORF1ab) and nucleocapsid protein(N), were simultaneously amplified using the real-time RT-PCR assay. The primers for *ORF1ab* gene were below: forward primer CCCTGTGGGTTTTACACTTAA; reverse primer: ACGATTGTGCATCAGCTGA; and the probe 5′-VIC-CCGTCTGCGGTATGTGGAAAGGTTATGG-BHQ1-3′. The primers for *N* gene were below: forward primer GGGGAACTTCTCCTGCTAGAAT; reverse primer CAGACATTTTGCTCTCAAGCTG; and the probe 5′-FAM-TTGCTGCTGCTTGACAGATT-TAMRA-3′. The real-time RT-PCR assay was performed using a 2019-nCoV nucleic acid detection kit according to the manufacturer's protocol (Shanghai bio-germ medical Technology Co, Ltd).Reaction mixture contains 12 μL of reaction buffer, 4 μL of enzyme solution, 4 μL of Probe primers solution, 3 μL of diethyl pyrocarbonate–treated water, and 2 μL of RNA template. RT-PCR assay was performed under the following conditions: incubation at 50 °C for 15 minutes and 95 °C for 5 minutes, 40 cycles of denaturation at 94°C for 15 seconds, and extending and collecting fluorescence signal at 55 °C for 45 seconds. A cycle threshold value (Ct-value) less than 38 was defined as a positive test result, and a Ct-value of 40 or more was defined as a negative test. These diagnostic criteria were based on the recommendation by the National Institute for Viral Disease Control and Prevention (China).

Table 1. Comparison of clinical features and outcomes of patients between CP treatment group and a non–CP treatment recent historic control group.

	CP treatment group (n=10)	Historic control group (n=10)	*p* value
**Demographics**			
Age, years	52.50 (45.00-59.50)	53.00 (46.50-60.50)	0.985
Gender			
Male	6 (60)	6 (60)	1.000
Female	4 (40)	4 (40)	
**Comorbidity**			
Yes	4 (40)	6 (60)	0.656
No	6 (60)	4 (40)	
**Baseline laboratory parameters**			
C-reactive protein	55.98 (15.57-66.67)	96.70 (33.92-173.39)	0.190
Lymphocyte	0.65 (0.53-0.90)	0.76 (0.54-1.32)	0.469
Alanine aminotransferase	42.00 (28.25-61.85)	20.35 (17.03-67.83)	0.133
Aspartate aminotransferase	38.10 (28.50-44.00)	37.35 (32.28-74.85)	0.764
Total bilirubin	12.40 (11.71-22.05)	8.70 (4.28-19.03)	0.065
SaO_2	93.00 (89.00-96.50)	93.00 (87.50-97.50)	0.923
**Clinical Outcome**			
Death	0(0)	3(30)	< 0.001*
Stable	0(0)	6(60)	
Improved	7(70)	1(10)	
Discharged	3(30)	0(0)	

Continuous variable (age) was expressed as median (IQR) and compared with Mann-Whitney U test. Categorical variables were expressed as n (%), where n was the total number of patients with available data, and compared by Fisher's exact test between convalescent plasma treatment group and control group.
